# 胸腔镜解剖性肺切除手术常见意外情况及其处置

**DOI:** 10.3779/j.issn.1009-3419.2016.06.17

**Published:** 2016-06-20

**Authors:** 建东 梅, 伦旭 刘

**Affiliations:** 1 610041 成都，四川大学华西医院胸外科 Department of Thoracic Surgery, West China Hospital, Sichuan University, Chengdu 610041, China; 2 610041 成都，中国西部肺癌早期诊断与综合治疗协同创新中心 Western China Collaborative Innovation Center for Early Diagnosis and Multidisciplinary Therapy of Lung Cancer, Chengdu 610041, China

**Keywords:** 胸腔镜, 解剖性肺切除, 意外情形, 中转开胸, 出血, Thoracoscope, Anatomical pulmonary resection, Unexpected situations, Conversion to thoracotomy, Bleeding

## Abstract

现代意义上的胸腔镜肺外科始于20世纪90年代初，经过20余年发展，手术技术已臻成熟，同时也有了丰富的临床数据积累。以胸腔镜为代表的微创技术在早期非小细胞肺癌及肺部良性疾病的外科治疗、肺部疾病诊断等方面的应用均已得到公认，也是上述情况的首选手术方法。随着胸腔镜解剖性肺切除手术的普及，文献中也逐渐有了一些临床实践中意外情形的报道，涉及解剖异常、病变自身相关因素、手术操作及技术等多方面，但目前少有对胸腔镜解剖性肺切除手术中意外情形的系统梳理，本文拟结合自身临床实践及文献报道，对这方面内容进行总结，为临床工作提供参考。

## Abstract (en)

The history of modern thoracoscopic pulmonary surgery could date back to the 1990s, and the related surgical technique has been matured after 20 years' development. Meanwhile, large amount of clinical data has been collected. Minimally invasive thoracic surgery represented by the thoracoscopic approach has been universally accepted as the preferred choice for the surgical treatment of early-staged non-small cell lung cancer and pulmonary benign diseases, and for the diagnosis of pulmonary diseases. With the generalization of thoracoscopic anatomical pulmonary resection, some unexpected situations during clinical practice has been reported in literatures, with issues involving anatomical variation, pathological factors, and surgical techniques. However, the systemic summary of the unexpected situations during thoracoscopic anatomical pulmonary resection is lacking until now. The present review, therefore, aims to summarize accidental issues and troubleshooting these unexpected situations on the basis of our own clinical practice and literature reports.

## 1 概述

自20世纪90年代初出现胸腔镜解剖性肺叶切除治疗非小细胞肺癌（non-small cell lung cancer, NSCLC）的报道^[1]^以来，以胸腔镜（Thoracoscope）为代表的微创技术在肺部疾病诊治中的应用被逐渐拓展，手术技术也日趋成熟，甚至还被用于涉及支气管及肺血管等结构重建的复杂成形手术^[2-4]^；与此同时，文献中也出现了一些胸腔镜解剖性肺切除手术中相关意外情况的报道，这其中最为直接的体现是对胸腔镜中转开胸原因的分析，常见原因包括血管损伤出血、邻近结构损伤、胸膜腔粘连、肺裂发育不全、肿瘤因素、淋巴结外侵、解剖变异等^[5-13]^，将上述中转开胸原因进行具体分类，大致可归为解剖因素、病理因素、手术操作技术性因素等方面，本文将结合我们自身临床实践中遇到的一些问题以及相关文献报道，对胸腔镜解剖性肺切除手术中各种可能的意外情况进行系统梳理，以期为临床提供参考。

## 2 解剖因素

### 2.1 肺裂发育不全

肺裂发育不全或未发育是肺手术中较为常见的解剖学变异。在胸腔镜解剖性肺切除手术过程中，对于肺动脉分支的处理，较为常用的方法之一是经叶间裂解剖并显露肺动脉，进而通过腔镜切割缝合器离断^[14]^，但对于叶间裂未发育或发育不全的患者，通过该方法游离肺动脉较为困难，这也使之成为不少中心开展胸腔镜肺叶切除手术时中转开胸的重要原因之一^[6, 11-13]^，同时，肺裂发育情况也是胸腔镜解剖性肺切除手术中转开胸的独立危险因素之一^[9]^。对于肺裂发育不全所造成的手术困难，可通过不经肺裂的胸腔镜肺叶切除方法加以克服^[15]^，最具代表性的方法即“单向式”胸腔镜肺叶切除^[16, 17]^，该方法仅在肺门解剖，依次离断血管及支气管，最后处理肺实质，我们目前尚未出现因肺裂发育问题所致的中转开胸^[18, 19]^。此外，Walker等^[20]^行上叶切除时所采用的“后路法”亦可避免应肺裂发育不全所致的肺上叶切除的操作不便。

### 2.2 血管变异

肺动脉分支数量及发出部位有较多变异，例如右肺动脉分支，少数情况下可有数支后升支动脉由肺动脉叶间段发出，甚至可由中叶动脉或背段动脉发出至上叶；肺静脉各分支的变异则相对较少，右肺中叶静脉通常汇入肺上静脉，少数情况下亦可汇入肺下静脉；此外，肺上静脉及肺下静脉还可能出现心包外共干的情形^[21]^。对于上述变异，术中解剖时加以仔细辨认，多可避免误伤或出血。我们以往曾经发生过左侧肺上及肺下静脉，因心包外共干被误断，后中转为开胸手术行静脉袖式成形^[18]^。需要格外加以重视的是肺隔离症，其往往存在血管变异，血供多来源于体循环，偶有隔离肺异常血管损伤导致出血^[19]^或因血管变异而中转开胸^[11]^的情形，对此，术前可仔细评估胸部增强计算机断层扫描（computed tomography, CT），确定隔离肺异常血管走行，必要时可行CT血管三维重建，术中再仔细加以辨认，避免将隔离肺的供血动脉误认为粘连带，从而导致严重出血。

## 3 病理因素

### 3.1 胸膜腔粘连/闭锁

胸膜腔粘连，尤其是胸膜腔闭锁，曾被认为是胸腔镜手术的禁忌症之一^[22]^，这也是胸腔镜解剖性肺切除手术中转开胸的一个常见原因^[5, 6, 12, 18]^。胸膜腔粘连限制了肺的萎陷，放置Trocar时易致肺的损伤，甚至因缺乏操作空间，致使腔镜手术难以实施；同时，游离胸膜腔粘连将延长手术时间，增加术中出血及肺损伤的可能。

对于胸腔镜肺手术中胸膜腔粘连的处理，王俊教授曾进行过一些总结，例如对条索状或局限性膜状粘连，可直接予以切断，但须注意对粘连带内血管的处理；对于弥漫性膜状粘连，可经手术切口进行钝性游离，其中一对切口的距离需小于10 cm，以便手指可在两切口间打通一个腔隙，再通过器械进行游离，直至可切除病变^[23]^。临床上术前常不易判断胸腔内是否存在粘连，为避免不必要的损伤，每台手术开始时均应注意这一问题。在作第一个切口，即观察孔时，进入胸膜腔前应嘱麻醉医师开始单肺通气，患侧气道开放，中弯钳钝性分离进入胸膜腔时若可闻及气体进入胸腔的声音，则多无严重粘连，否则需考虑到胸膜腔广泛粘连的可能，应加以核实。对于胸腔内粘连带或局限性粘连，处理相对简单；若证实为胸膜腔广泛粘连，我们通常先以手指经观察孔进行钝性分离，如能成功进行钝性游离并置入胸腔镜观察，则可继续作操作孔切口，以同样的方法在操作孔周围进行钝性分离，进而借助器械（如环钳）与手指共同进行钝性分离，在各孔之间建立隧道，可为胸腔镜操作提供空间，再以电凝钩及吸引器配合，游离粘连；如若无法通钝性分离进入胸腔或建立隧道，则提示粘连较为致密，通常需转为开胸手术，此即处理胸膜腔粘连的“隧道法指征”。

### 3.2 肺门结构致密粘连

肺门结构如支气管与动脉之间致密粘连也是术中常见的困难意外情形之一，这也是胸腔镜解剖性肺切除手术中转开胸的常见原因^[5, 6, 11-13]^。我国结核发病率有上升趋势，在结核高发地区，患者存在肺门致密粘连的情况更为常见。除结核等慢性炎症之外，接受术前新辅助治疗者也常存在肺门组织纤维化、致密粘连。对于影像学提示肺门或纵隔钙化者，合并肺门致密粘连的几率较大，通过对胸腹部、肺门及纵隔钙化灶的评估，可预测胸腔镜解剖性肺切除手术中转开胸的可能性^[7]^。

对肺门致密粘连的患者，肺静脉一般都能够顺利游离出来，常见的问题往往是肺动脉与支气管之间存在致密粘连淋巴结，解剖过程中可能出现肺动脉损伤出血，对此，我们最常采取的措施是采用“预阻断”策略，即一旦发现肺动脉与支气管间解剖困难时，改为游离肺动脉近心端，以胸腔镜下可释放的无损伤血管阻断钳阻断肺动脉近心端，再继续解剖，如此使困难解剖变得容易，即便出现损伤，出血亦在控制之下，保证了手术的安全性^[24]^。第二个策略是在离断肺静脉后，如遭遇支气管与肺动脉致密粘连，此时可直接切开支气管，为肺动脉处理让出空间，在处理肺动脉后再闭合支气管残端，此种情况主要出现于左上肺叶切除。第三种策略为支气管肺动脉同时切割缝合，常用于因结核性淋巴结钙化包裹支气管肺动脉，使其融为一体；此时用厚组织钉一同离断叶支气管和叶肺动脉，然后其残端用3-0 Prolene线间断褥式缝合加固。

### 3.3 肿瘤/肿瘤转移淋巴结外侵

肿瘤或转移淋巴结外侵多可通过术前影像学评估发现，设计手术方式时加以考虑，偶有术中发现因肿瘤或转移淋巴结外侵，需改为开胸手术的病例，包括肿瘤累及胸壁、累及肺门结构或纵隔大血管等^[7, 11, 18, 25, 26]^，术中发现此类情形时，需结合自身腔镜下操作的能力，无需强求全腔镜下完成手术，必要时尽早中转开胸。对于部分患者，如肿瘤累及上腔静脉，亦可尝试腔镜下行上腔静脉部分切除成形^[27]^。中央型肺癌或肺门转移淋巴结侵及支气管或肺动脉时，对于经验丰富的中心，可尝试在胸腔镜下行支气管/肺动脉成形^[3, 28-31]^，我们早在2012年即开始探索胸腔镜双袖式成形肺叶切除治疗有局部外侵的中央型肺癌^[2]^。

## 4 操作技术性因素

由于各种各样操作原因所致的意外情况，在手术中相对更为常见，应尽量通过提高操作技能加以避免，其中最为紧急的当属血管意外损伤所致的出血，其他还包括术野渗血、切口出血、邻近结构损伤等，在此分别加以描述。

### 4.1 手术创面渗血

手术中解剖肺门结构或进行淋巴结清扫时，部分术者习惯于通过钝性分离或剪刀锐性解剖，其主要弊端在于引起手术创面较多渗血，影响术野清晰度，可能造成解剖结构的辨认不清，甚或误伤。对此，我们的解决方案是采用吸引器与电钩配合，以吸引器暴露，同时清除烟雾及术野出血，电凝钩切开组织的同时止血，形成了“吸引-电凝无血化游离技术”，操作过程中保证了术野的清洁，减少出血，且利于显露，进行纵隔淋巴结清扫的时候，可以超声刀代替电凝钩，同样可达到无血化的解剖。

### 4.2 手术切口出血

手术切口出血是胸腔镜手术中较为常见的问题之一，切口出血可能影响术野的显露，或导致镜头模糊，甚至有少数患者术后因切口出血需再次手术探查止血。对此，作胸腔镜观察孔及操作孔时应尽量沿肋骨上缘进胸，同时对切口内组织予以充分止血；其次，通过放置Trocar及切口保护套，减少器械进出胸腔时对切口的扰动，减少出血机会；第三，关胸前应仔细检查切口有无渗血，并给予相应处理后方可关胸。

### 4.3 大血管损伤出血

术中各种原因所致的大血管损伤出血无疑是胸腔镜解剖性肺切除手术最为棘手且紧急的意外情况，尤其是肺动脉、上腔静脉等大血管损伤出血更是如此，其中以肺动脉损伤出血较为常见^[19, 32, 33]^。在文献报道中，血管损伤出血也是胸腔镜解剖性肺切除手术中转开胸最为常见的一个原因^[5-8, 11, 13, 25, 26, 34-41]^，部分中心的资料中血管损伤出血甚至占了所有中转开胸病例的三分之一以上^[6, 11, 25, 26, 33, 35-37]^。导致大血管意外损伤的具体原因包括锐性解剖损伤、切割缝合器撕裂、钝性解剖时撕裂、合成夹或线结脱落、能量器械损伤等^[19, 33, 42]^。对这一问题的处置，最为重要的是注意防范血管损伤，术前需加强对血管变异、致密粘连等可能导致血管损伤情形的评估，如术中发现血管解剖困难时，可采用前文提及的“预阻断”策略处理，必要时尽早改为开胸手术，以避免发生难以控制的出血。

对于已经发生的血管损伤出血，首要的问题是保持镇静，避免慌乱中钳夹或缝合，导致破口进一步扩大，使出血愈加不可控。我们设计了“吸引-侧压止血技术”（suction-compressing angiorrhaphy technique, SCAT）进行处理，约90%的血管损伤出血通过该方法成功处理，无需中转开胸，而且出血不多^[19]^。我们在手术过程中采用“吸引-电凝无血化游离技术”进行解剖，吸引器头端带有侧孔，一旦有出血发生，可及时以吸引器通过侧壁压迫的方法控制住出血，同时可借助吸力清除积血，清楚显露出血部位；如吸引器不在胸腔内时，一旦发生出血，吸引器置入胸腔也较其他器材如纱布等方便快捷。如出血量很大，需马上再借助另一吸引器清除积血。一旦出血被控制，则下一步评估血管破口的部位及大小，小于5 mm的破口，通常可借助吸引器压迫损伤部位，以5-0 Prolene线在破口两端直接缝合后打结（我们称之为滚动缝合）；大于5 mm但不超过血管管径1/3的破口，可在吸引器控制出血后，以Allis钳轻轻钳夹破口，移除吸引器后再以Prolene线双重连续缝合修补破口（我们称之为钳夹缝合）；若破口超过管径1/3或Allis钳阻碍缝合，则需游离血管近心端并加以阻断，再以Prolene线连续缝合修补破口（我们称之为阻断缝合）^[19, 43]^。

近年来，随着胸腔镜手术技术的逐渐成熟，其他一些中心也分享了他们处理胸腔镜解剖性肺切除手术中血管损伤出血的一些经验。Yamashita等^[33]^总结了26例胸腔镜手术中血管损伤出血的处理，半数中转为开胸手术，6例改为胸腔镜辅助小切口处理，其他一些小的出血则主要依靠止血材料处理。Miyazaki^[32]^对胸腔镜术中血管损伤的处理则主要采用纱布棒或肺实质压迫止血，尽量保持腔镜视野清晰，进而尝试采用止血材料（TachoSil^®^）覆盖破口，并采用纱布压迫数分钟，两次尝试无效后则中转为开胸手术。此外，如出血难以腔镜下处理，可通过压迫暂时控制出血，为中转开胸争取时间^[34, 42]^。

需要强调的是胸腔镜下大出血的处理，需以患者安全为第一宗旨，如评估后术者认为无法在胸腔镜下控制出血，需尽快中转为开胸手术，中转开胸并不意味着手术失败。

### 4.4 乳糜漏

胸腔镜肺切除手术中偶可出现胸导管或其分支损伤，导致术后乳糜胸，多由于清扫淋巴结所致，以右侧手术居多，发生率约2.5%^[44-46]^。为预防术后乳糜漏，清扫淋巴结时应注意对管道样结构的处理，尤其是清扫2、4组淋巴结时，对于由淋巴结内发出的管道样结构，应以超声刀或钛夹仔细处理，如术中发现明显乳糜漏，必要时可预防性结扎胸导管。

### 4.5 血管或支气管误断

少数情况下，胸腔镜解剖性肺叶切除过程中可能出现非病变肺叶血管或支气管被误断的情况。Decalume等^[35]^汇总了欧洲6个中心共3, 076例胸腔镜解剖性肺切除手术的资料，其中3例行右肺上叶切除的患者肺动脉远端被误断，2例右肺上叶切除手术中误断中间支气管，1例左肺上叶切除术中于心包内将肺上静脉及肺下静脉一并切断，1例右肺上叶切除时误断中叶静脉。导致上述误伤的主要原因多在于术中对肺门结构的显露不佳，从而引起误判，因而术中离断肺门结构前应进行充分确认，如难以较好显露或一旦出现误伤，应及时中转开胸，以减少对患者的损伤。

### 4.6 肺扭转

无论开胸手术或胸腔镜手术，在关胸后均有发生肺扭转的可能，右肺中叶因体积较小，易出现扭转，如能早期发现，尚可通过胸腔镜手术予以复位，如肺叶明显梗死，则需行肺叶切除^[47-49]^。近年来还有人观察到上叶切除术中常规离断肺下韧带可能增加肺扭转的发生，因而提出保留肺下韧带，但该观点并未得到广泛认可^[50]^。对术后肺扭转的问题，关键应在于预防，关胸时应在胸腔镜观察下进行肺复张，确认残余肺叶复张良好且位置正常，其后可维持双肺通气。对过于游离的肺叶，可将残留的两个肺叶作适当缝合，防止肺肺扭转的发生。

### 4.7 其他组织器官损伤

胸腔镜肺手术中还可能出现其他一些重要结构的损伤，例如喉返神经、气管、食管、膈肌、甚至脾脏等^[35, 44, 46]^。其中喉返神经损伤多由于清扫淋巴结时所致。淋巴结清扫时能量器械的使用亦可造成食管或气管的热损伤。此外，偶有气管插管球囊过度充气致气管撕裂的报道^[8, 35]^。Decalume等^[35]^报道的资料中还包含1例切割缝合器致食管损伤的病例，因切割缝合器前端夹闭部分食管致其损伤，因而，在放置切割缝合器时应注意检查有无副损伤。左胸手术时对膈肌的按压也有伤及脾脏的可能。

## 5 其他

### 5.1 术中病变定位困难

随着低剂量螺旋CT筛查的推广，以肺部小结节，特别是磨玻璃样结节为主要表现的早期肺癌，在手术病例中有增加趋势。对于肺内小结节的定位，术前可通过CT引导下穿刺定位^[51]^，注射硬化剂或染料，放置弹簧圈等。但少数情况下可能出现定位困难的情况，例如定位钢丝脱落或术前未能穿刺定位等，对于这种情况，如术中难以扪及结节，可尝试通过术中超声对肺内结节进行定位^[52]^。我们在肺结节手术中常采用“缝线定位法”，即采用腔镜肺钳经副操作孔将肺叶向主操作孔牵拉，经主操作孔以手指进行触诊，扪及结节后以缝线在相应肺表面标记，再依此标记行局部切除^[53]^。我们的经验，≥7 mm的结节距肺表面2 cm之内，术中都能通过手指扪及结节。此外，极少数情况下，术前未做穿刺定位，通过上述方法定位时手指无法扪及结节，在无其他办法的情况下，可根据解剖标志进行定位，即测量结节至纵隔面、肺尖或叶间裂的距离，以此判断结节所在的大致部位，再以缝线标记后行亚肺叶切除，切开肺组织寻找病灶。

### 5.2 切割缝合器机械故障

目前临床所用的腔镜切割缝合器有较好的可靠性，尽管如此，依然偶有出现机械故障的时候，例如切割缝合器切割肺组织时被卡住，无法松开的情形。避免这一情形的重点是在放置切割缝合器时，拟切割组织中勿含有合成夹、钛夹等异物，钉夹中勿残留前次切割的缝合钉；还应根据拟切割的组织厚度选择合适的钉仓，避免成钉后爆裂、出血或漏气等情形。

## 6 小结

胸腔镜解剖性肺切除手术已逐渐成熟，但只有对各种术中意外情况有充分的认识，才能尽量避免其发生。本文依据各种意外情形发生的原因不同，大致将其归为解剖变异、病理因素及手术操作相关的意外情况，其中解剖变异及手术操作相关的意外情况，多数可通过术前仔细评估、术中保持良好的显露及清晰的术野加以避免。各类手术意外情形中最为紧急与严重的情况当属大血管损伤所致的出血，处理这一情况时应视自身技术熟练程度，以安全为第一位，对于出血较多或腔镜下处理困难时应及时中转开胸，中转开胸并不意味着手术失败。
